# Epidemiological and clinical characteristics of early COVID-19 cases, United Kingdom of Great Britain and Northern Ireland

**DOI:** 10.2471/BLT.20.265603

**Published:** 2020-11-30

**Authors:** Nicola L Boddington, Andre Charlett, Suzanne Elgohari, Chloe Byers, Laura Coughlan, Tatiana Garcia Vilaplana, Rosie Whillock, Mary Sinnathamby, Nikolaos Panagiotopoulos, Louise Letley, Pauline MacDonald, Roberto Vivancos, Obaghe Edeghere, Joseph Shingleton, Emma Bennett, Simon Cottrell, Jim McMenamin, Maria Zambon, Mary Ramsay, Gavin Dabrera, Vanessa Saliba, Jamie Lopez Bernal

**Affiliations:** a Public Health England, 61 Colindale Avenue, London, NW9 5EQ, England.; b Public Health England, Liverpool, England.; c Public Health England, Salisbury, England.; d Public Health Wales, Cardiff, Wales.; e Health Protection Scotland, Glasgow, Scotland.

## Abstract

**Objective:**

To describe the clinical presentation, course of disease and health-care seeking behaviour of the first few hundred cases of coronavirus disease 2019 (COVID-19) in the United Kingdom of Great Britain and Northern Ireland.

**Methods:**

We implemented the World Health Organization’s First Few X cases and contacts investigation protocol for COVID-19. Trained public health professionals collected information on 381 virologically confirmed COVID-19 cases from 31 January 2020 to 9 April 2020. We actively followed up cases to identify exposure to infection, symptoms and outcomes. We also collected limited data on 752 symptomatic people testing negative for COVID-19, as a control group for analyses of the sensitivity, specificity and predictive value of symptoms.

**Findings:**

Approximately half of the COVID-19 cases were imported (196 cases; 51.4%), of whom the majority had recent travel to Italy (140 cases; 71.4%). Of the 94 (24.7%) secondary cases, almost all reported close contact with a confirmed case (93 cases; 98.9%), many through household contact (37 cases; 39.8%). By age, a lower proportion of children had COVID-19. Most cases presented with cough, fever and fatigue. The sensitivity and specificity of symptoms varied by age, with nonlinear relationships with age. Although the proportion of COVID-19 cases with fever increased with age, for those with other respiratory infections the occurrence of fever decreased with age. The occurrence of shortness of breath also increased with age in a greater proportion of COVID-19 cases.

**Conclusion:**

The study has provided useful evidence for generating case definitions and has informed modelling studies of the likely burden of COVID-19.

## Résumé

**Objectif:**

Décrire la manifestation clinique, l'évolution de la maladie et la propension à recourir aux services de santé des premières centaines de cas de maladie à coronavirus 2019 (COVID-19) au Royaume-Uni de Grande-Bretagne et d'Irlande du Nord.

**Méthodes:**

Nous avons appliqué le *Protocole d'enquête sur la maladie à coronavirus 2019 (COVID-19) – les X premiers cas et contacts (FFX)* publié par l'Organisation mondiale de la Santé. Des professionnels de la santé formés à cet effet ont collecté des informations sur 381 cas virologiquement confirmés de COVID-19 entre le 31 janvier 2020 et le 9 avril 2020. Nous avons étroitement surveillé ces cas afin d'identifier la source d'exposition à l'infection, les symptômes observés et l'issue de la maladie. Nous avons également récolté des données sur 752 personnes symptomatiques testées négatives à la COVID-19 qui ont servi de groupe de contrôle pour analyser la sensibilité, la spécificité et la valeur prédictive des symptômes.

**Résultats:**

Environ la moitié des cas de COVID-19 ont été importés (196 cas; 51,4%), la majorité s'étant récemment rendue en Italie (140 cas; 71,4%). Sur les 94 (24,7%) cas secondaires, presque tous ont signalé un contact rapproché avec un cas confirmé (93 cas; 98,9%), généralement au sein de leur foyer (37 cas; 39,8%). Au niveau de l'âge, la proportion d'enfants ayant contracté la COVID-19 était moindre. La plupart des cas avaient de la toux, de la fièvre et de la fatigue. La sensibilité et la spécificité des symptômes variaient en fonction de l'âge, présentant une relation non linéaire avec cet élément. Plus l'âge des cas de COVID-19 avançait, plus ils étaient susceptibles de développer de la fièvre, à l'exception de ceux touchés par d'autres infections respiratoires. Le risque d'essoufflement augmentait lui aussi avec l'âge chez une grande partie des cas de COVID-19.

**Conclusion:**

Cette étude a apporté un éclairage utile dans l'établissement d'une définition des cas. Elle a également fourni, pour diverses modélisations, des indications sur la charge que pourrait faire peser la COVID-19.

## Resumen

**Objetivo:**

Describir la presentación clínica, el curso de la enfermedad y el comportamiento de búsqueda de atención sanitaria de los primeros cientos de casos de la enfermedad del coronavirus de 2019 (COVID-19) en Reino Unido e Irlanda del Norte.

**Métodos:**

Implementamos el protocolo de investigación de los primeros casos y contactos de la Organización Mundial de la Salud para la COVID-19. Profesionales expertos en el área de la salud pública recopilaron información sobre 381 casos virológicamente confirmados de COVID-19 entre el 31 de enero de 2020 y el 9 de abril de 2020. Realizamos un seguimiento activo de los casos para identificar la exposición a la infección, los síntomas y los resultados. También recogimos datos limitados sobre 752 personas sintomáticas que dieron negativo en los resultados de la COVID-19, como grupo de control para el análisis de la sensibilidad, la especificidad y el valor predictivo de los síntomas.

**Resultados:**

Casi la mitad de los casos de COVID-19 fueron importados (196 casos; 51,4%), de los cuales la mayoría había hecho un viaje reciente a Italia (140 casos; 71,4%). De los 94 (24,7%) casos secundarios, casi todos informaron de un contacto cercano con un caso confirmado (93 casos; 98,9%), muchos de ellos a través del contacto con el hogar (37 casos; 39,8%). Por edad, una proporción menor de niños tenía COVID-19. La mayoría de los casos se presentaban con tos, fiebre y fatiga. La sensibilidad y especificidad de los síntomas variaba según la edad, con relaciones no lineales con la edad. Aunque la proporción de casos de COVID-19 con fiebre aumentaba con la edad, en el caso de los que tenían otras infecciones respiratorias la aparición de fiebre disminuía con la edad. La incidencia de la falta de aliento también aumentó con la edad en una mayor proporción de casos de COVID-19.

**Conclusión:**

El estudio ha aportado pruebas útiles para generar definiciones de casos y también ha servido de base a los estudios de modelización del probable coste de la COVID-19.

## ملخص

الغرض وصف العرض السريري، ومسار المرض وسلوك البحث عن الرعاية الصحية لأول بضع مئات من حالات الإصابة بمرض فيروس كورونا 2019 (كوفيد 19) في المملكة المتحدة لبريطانيا العظمى وأيرلندا الشمالية.

الطريقة قمنا بتنفيذ بروتوكول منظمة الصحة العالمية للتحقيق بخصوص الحالات الـ X القليلة الأولى للإصابة بكوفيد 19، والمخالطين لهم. وقام أخصائيو الصحة العامة المدربون بتجميع معلومات عن 381 حالة إصابة فيروسية مؤكدة بكوفيد 19 في الفترة من 31 يناير/كانون ثاني 2020 إلى 9 أبريل (نيسان) 2020. وتابعنا بدأب الحالات لتحديد التعرض للعدوى والأعراض والنتائج. كما قمنا بجمع بيانات محدودة عن 752 شخصاً يعانون من أعراض كوفيد 19، ولكن كانت نتيجة الاختبار سلبية، كمجموعة تحكم لتحليلات الحساسية، والنوعية، والقيمة التنبؤية للأعراض.

النتائج تم استيراد نصف حالات الإصابة بكوفيد 19 تقريباً (196 حالة؛ 51.4%)، سافر معظمهم مؤخراً إلى إيطاليا (140 حالة؛ 71.4%). من بين 94 حالة ثانوية (24.7%)، أبلغت كل الحالات تقريباً عن مخالطة وثيقة مع حالة مؤكدة (93 حالة؛ 98.9%)، والعديد منها عن طريق الاتصال المنزلي (37 حالة؛ 39.8%). وبالنسبة للعمر، فقد أُصيبت نسبة أقل من الأطفال بكوفيد 19. وكانت معظم الحالات مصحوبة بسعال وحمى وإرهاق. تختلف حساسية ونوعية الأعراض تبعاً للعمر، مع علاقات غير خطية مع العمر. على الرغم من أن نسبة حالات الإصابة بكوفيد 19 المصحوبة بالحمى قد زادت مع تقدم العمر، إلا أنه بالنسبة للمصابين بأمراض الجهاز التنفسي، فإن الإصابة بالحمى انخفضت مع التقدم في العمر. كما زاد كذلك حدوث ضيق التنفس مع التقدم في العمر في نسبة أكبر من حالات الإصابة بكوفيد 19.

الاستنتاج قدمت الدراسة أدلة مفيدة لوضع تعريفات الحالة، وأوضحت لدراسات النماذج العبء المحتمل لمرض كوفيد 19.

## 摘要

**目的:**

旨在描述大不列颠及北爱尔兰联合王国最初数百个新型冠状病毒肺炎（新冠肺炎）病例的临床表现、病程及就医行为。

**方法:**

我们实施了世界卫生组织制定的少数最初新冠肺炎病例和接触者调查方案。经过培训的公共卫生专业人员收集了 2020 年 1 月 31 日至 2020 年 4 月 9 日期间通过病毒学方法确诊的 381 例新冠肺炎病例的相关信息。我们积极跟踪病例，以确定接触感染情况、症状和结果。我们还针对 752 名具有新冠肺炎症状但其检测结果为阴性的病例收集了有限的数据，并将其作为对照组，以分析症状的敏感性、特异性和预测价值。

**结果:**

约有一半的新冠肺炎病例属于境外输入病例（196 例；51.4%），且其中大多数病例近期曾前往意大利旅行（140 例；71.4%）。在 94 例 (24.7%) 二代病例中，几乎所有病例都报告称曾与确诊病例密切接触（93 例；98.9%），且许多病例是因家庭内部接触而导致感染（37 例；39.8%）。按年龄统计，患有新冠肺炎的儿童比例较低。大多数病例表现为咳嗽、发热和疲乏。症状的敏感性和特异性因年龄而异，并与年龄呈非线性关系。虽然其他呼吸道疾病感染病例的发热症状发生率随着年龄增长而降低，但新冠肺炎病例的发热症状发生比例却随年龄增长而增加。而且，大部分新冠肺炎病例的呼吸短促发生率也随年龄的增长而增加。

**结论:**

本研究为制定诊断标准提供了有用的证据，并为针对新冠肺炎潜在负担的模拟研究提供了素材。

## Резюме

**Цель:**

Описать клинические признаки, течение болезни и обращение за медицинской помощью первых нескольких сотен случаев коронавирусного заболевания 2019 г. (COVID-19) в Соединенном Королевстве Великобритании и Северной Ирландии.

**Методы:**

Авторы внедрили протокол исследования первых случаев заболевания новой коронавирусной инфекцией 2019 г. (COVID-19) и контактов, разработанный Всемирной организацией здравоохранения. Квалифицированные специалисты в области общественного здравоохранения собрали информацию о 381 подтвержденном вирусологическим тестированием случае заболевания COVID-19 в период с 31 января по 9 апреля 2020 года. Авторы активно отслеживали данные случаи для определения контакта с инфекцией, изучения симптомов и исхода болезни. Были собраны ограниченные данные по 752 пациентам, имеющим клинические симптомы заболевания, но отрицательный результат теста на COVID-19, в качестве контрольной группы для анализа чувствительности, специфичности и прогностической ценности симптомов.

**Результаты:**

Примерно половина случаев COVID-19 была завезена из-за границы (196 случаев; 51,4%), большинство из которых недавно путешествовали в Италию (140 случаев; 71,4%). Из 94 (24,7%) вторичных случаев заражения почти все сообщали о тесном контакте с лицами с подтвержденным случаями заболевания (93 случая; 98,9%), многие из которых проживали совместно с инфицированными (37 случаев; 39,8%). Вирус COVID-19 редко заражает детей. В большинстве случаев отмечались кашель, высокая температура и утомляемость. Чувствительность и специфичность симптомов варьировались в зависимости от возраста и находились в нелинейной зависимости от возраста. Несмотря на то что доля случаев COVID-19 с высокой температурой увеличивалась с возрастом, у людей с другими респираторными инфекциями частота наличия лихорадки с возрастом снижалась. Возникновение одышки также увеличивалось с возрастом для большинства случаев COVID-19.

**Вывод:**

Исследование предоставило полезную информацию для определения случаев возникновения и послужило основой для модельных исследований вероятного бремени COVID-19.

## Introduction

The World Health Organization (WHO) recommended that Member States implement their established First Few X enhanced surveillance protocol^1^ to investigate the clinical and epidemiological characteristics of at least the first 100 confirmed coronavirus disease 2019 (COVID-19) cases and their close contacts.^2^ The design was used in the 2009 influenza H1N1 pandemic.^3^ Following the detection of the first laboratory-confirmed COVID-19 cases in the United Kingdom of Great Britain and Northern Ireland at the end of January 2020, Public Health England – the national public health agency in England – initiated the First Few X surveillance system for COVID-19.

The epidemiology and clinical features of early COVID-19 cases identified in China and elsewhere have previously been reported.^4^^–^^9^ A pooled analysis of 1155 cases from seven countries provided estimates of key epidemiological parameters^10^ and the first cases identified in the WHO European Region have been described.^11^ The most commonly reported symptoms were fever, fatigue, dry cough, myalgia and dyspnoea.^4^^–^^7^^,^^10^^–^^12^ However, these studies did not report on the sensitivity, specificity or positive predictive values of symptoms.

The United Kingdom was one of the first countries affected in Europe, with its first two confirmed cases of COVID-19 detected on 31 January 2020.^11^ For this study we describe the epidemiological and clinical characteristics of the first few hundred cases of COVID-19 identified in the country, including estimates of sensitivity and specificity of selected symptoms. We describe implementation of the WHO First Few X protocol for COVID-19 and discuss some of the lessons learnt and how the data informed the public health response to COVID-19 in the United Kingdom.

## Methods

Following reports of the COVID-19 epidemic in China, staff at Public Health England modified the existing pandemic influenza First Few X protocol for the COVID-19 outbreak, including the data collection questionnaires and electronic data capture system. Data was collected between 31 January 2020 and 9 April 2020. The process was guided by the First Few COVID-19 X cases and contacts transmission investigation protocol.^1^ Cases from England, Scotland and Wales were reported to the FF100 surveillance system.

### Case ascertainment

Case definitions for testing and the time periods that they applied are outlined in Box 1. Initially we recruited all people in the United Kingdom with virologically confirmed COVID-19. However, due to the large predominance of imported cases during February 2020, we later restricted recruitment to sporadic cases only.

Box 1 **Summary of case definitions of COVID-19 for population testing in the United Kingdom of Great Britain and Northern Ireland at different time periods of 2020**

**Before 7 February**

Epidemiological criteria: In the 14 days before the onset of illness, travel to China, OR contact with a confirmed case of COVID-19 (previously referred to as 2019-nCoV infection);

AND

Clinical criteria: Severe acute respiratory infection requiring admission to hospital with clinical or radiological evidence of pneumonia or acute respiratory distress syndrome, OR acute respiratory infection of any degree of severity, including at least one of shortness of breath (difficult breathing in children) or cough (with or without fever), OR fever with no other symptoms.

**From 7 February**

Epidemiological criteria: In the 14 days before the onset of illness, travel to affected countries (the list of affected countries was expanded between 7 February and 13 March 2020), including transit, for any length of time, in these countries, OR contact with confirmed cases of COVID-19 (previously referred to as 2019-nCoV infection);

AND

Clinical criteria: Severe acute respiratory infection requiring admission to hospital with clinical or radiological evidence of pneumonia or acute respiratory distress syndrome, OR acute respiratory infection of any degree of severity, including at least one of shortness of breath (difficult breathing in children) or cough (with or without fever), OR fever with no other symptoms.

**From 13 March**

Inpatient definition: Patient requiring admission to hospital (a hospital practitioner has decided that admission to hospital is required with an expectation that the patient will need to stay at least one night);

AND

Patient has either clinical or radiological evidence of pneumonia, OR acute respiratory distress syndrome, OR influenza-like illness defined as fever 37.8 °C and at least one of the following respiratory symptoms, which must be of acute onset: persistent cough (with or without sputum), hoarseness, nasal discharge or congestion, shortness of breath, sore throat, wheezing, sneezing.

COVID-19: coronavirus disease 2019; United Kingdom: United Kingdom of Great Britain and Northern Ireland.

We defined imported cases as people with travel to countries with known COVID-19 circulation at the time or people having contact with a confirmed case while abroad within 14 days of the onset of their own symptoms. Secondary cases were defined as people who had contact with a confirmed case or a probable or suspected case in the United Kingdom and did not fit the definition of an imported case. Sporadic cases were people with no travel history to countries with known COVID-19 circulation, and no known contact with a confirmed case.

As part of the First Few X protocol, we identified and followed up close contacts of confirmed cases. Due to the large numbers of contacts, we restricted follow-up to close contacts, including people in the household; other people with direct face-to-face contact; and health-care workers who had not worn recommended personal protective equipment. The results of the close-contact follow-ups are described elsewhere.^13^

### Data collection

On identification of a positive case, staff from the local Public Health England teams (or the equivalent in the devolved administrations of Wales and Scotland) were asked to collect information about the person. The teams used an initial enhanced surveillance questionnaire to record information on the infected person’s demographic details, medical history and travel history. The information was collected as soon as possible after a positive laboratory result was reported, through interview with the infected person or, if the person was too unwell or had died, with a health-care worker or family member.

We followed up cases after 14 days from the initial report. Follow-up information on cases was collected to determine the patient’s clinical outcome and the occurrence of any medical complications. To improve completeness of the initial questionnaires and to achieve a high rate of follow-up, we trained a team of health protection practitioners, nurses, doctors and field epidemiologists to proactively follow up the cases in England using telephone interviews. The data collected on underlying health conditions are presented elsewhere.^14^ We entered the data from completed forms into a dedicated First Few X secure web database to extract, clean and quality-check the data.

To analyse the predictive values of respiratory symptoms we used data collected in the early stages of the epidemic by local health protection teams on all possible cases of COVID-19. The questionnaires comprised a minimum data set, including patient demographics, presenting illness (cough, fever, sore throat and shortness of breath), clinical course or complications after onset, and exposure to possible infection in the 14 days before onset of first symptoms. We used data on suspected cases with respiratory symptoms who tested negative for COVID-19 as a control group for the analyses of symptoms (mostly only tested once).

Relevant anonymized data is available through the Public Health England Office for Data Release, Public Health England, United Kingdom.^15^

### Data analysis

We made descriptive analyses of the COVID-19 study cases in relation to patient characteristics, clinical symptoms and complications, health-care interactions and outcomes. For the analysis of symptoms, we assumed that missing data indicated absence of that symptom. We assigned ethnicity to cases by linking to the Hospital Episode Statistics database, a national database of all hospital admissions, emergency department attendances and outpatient appointments.^16^

We estimated the sensitivity and specificity of respiratory symptoms using data on symptoms from positive and negative cases of COVID-19. The positive cases were those with laboratory-confirmed COVID-19 from the First Few X study. The negative cases were symptomatic people who were confirmed negative for COVID-19 in the minimum data set. We calculated sensitivity as the proportion of positive cases who had a specific symptom, among those people selected for testing, and specificity as the proportion of those who tested negative who did not have a specific symptom, among those selected for testing. We estimated predictive values for the observed prevalence of COVID-19 positive patients. The positive predictive value was determined as the probability of those people with a specific symptom testing positive, and the negative predictive value as the probability of those without a specific symptom testing negative.

We explored the functional relationships between the presence of a symptom and the patient’s age using locally weighted scatter plot smoothing using the proportion of positive and negative cases. We used fractional polynomial logistic regression models to obtain parametric functions of these relationships with age, capturing the nonlinear relationships between the presence of symptoms and age. We used interaction terms between age and case type (imported, sporadic or secondary) to assess if there was evidence of different age relationships.

We performed logistic regression analyses to assess which symptoms were independently associated with COVID-19, accounting for sex and age. We modelled age as a continuous variable: the estimated average change in odds for a 10-year increase in age. We used multinomial regression models with case type as the outcome variable to assess whether the associations with symptoms differed for each case type. We used a simplified categorization of age in three broad age groups. 

We undertook analyses using Microsoft Excel 2010 (Microsoft Corp., Redmond, United States of America, USA), R version 3.5.0 (R Foundation, Vienna, Austria)^17^ and Stata 16 MP (StataCorp, College Station, USA).

### Ethical considerations

This was an observational surveillance system carried out under the permissions granted under regulation 3 of the United Kingdom Health Service (Control of Patient Information) Regulations 2002, and without explicit patient permission under Section 251 of the National Health Service Act 2006.

## Results

**Fig. 1:**
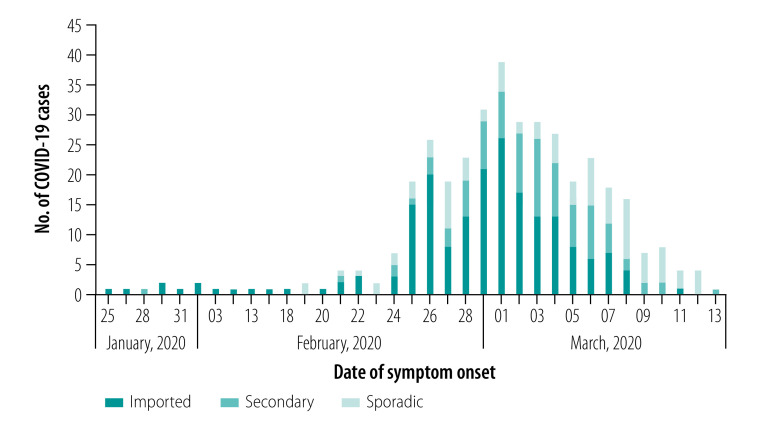
Date of symptom onset and case type of COVID-19 cases in the First Few X study in the United Kingdom of Great Britain and Northern Ireland, 31 January to 9 April 2020

**Fig. 2:**
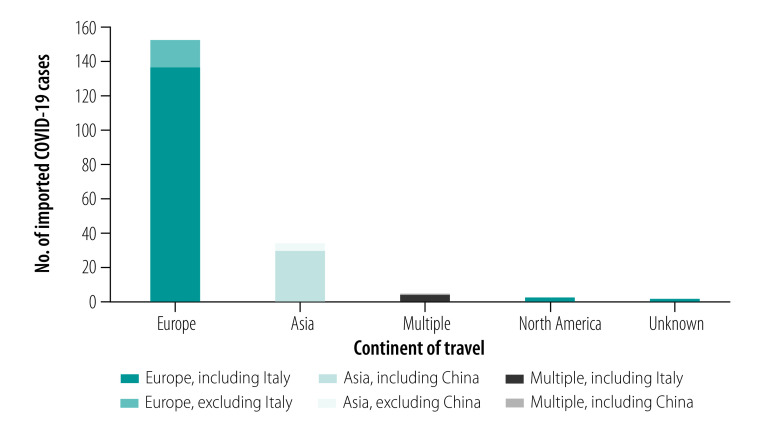
Continent of travel in 14 days before symptom onset among imported COVID-19 cases in the First Few X study in the United Kingdom of Great Britain and Northern Ireland, 31 January to 9 April 2020

We included 381 confirmed cases of COVID-19 from 31 January 2020 up to 9 April 2020 in the study: 359 cases from England, 19 from Scotland and three from Wales. Fig. 1 shows the distribution of cases by date of symptom onset and COVID-19 case types. Approximately half of the 381 cases were imported (196; 51.4%) with the remainder being secondary (94; 24.7%) or sporadic (91; 23.9%) cases. Of the 196 imported cases, 140 (71.4%) patients reported travel to Italy in the 14 days before symptom onset and hence Europe was the most commonly visited continent by the COVID-19 infected patients (Fig. 2). Where occupation was recorded (357 cases), 42 patients (11.8%) were health-care workers, the majority of whom (26 cases) were imported cases. Of the 94 secondary cases, almost all patients (93; 98.9%) reported close contact with a confirmed case: 37 (39.8%) had close contact within a household setting, 10 (10.8%) in a health-care setting, 44 (47.3%) in other settings (for example work setting, social gatherings) and 3 (3.2%) in an unknown setting.

COVID-19: coronavirus disease 2019.

Note: Data on date of symptom onset were available for 375 cases of confirmed COVID-19.

COVID-19: coronavirus disease 2019.

Note: Data were available for 196 imported cases with confirmed COVID-19.

**Fig. 3:**
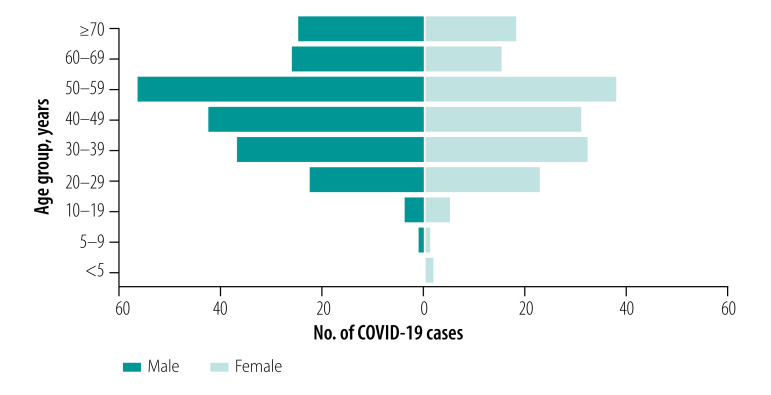
Age and sex distribution of COVID-19 cases in the First Few X study in the United Kingdom of Great Britain and Northern Ireland, 31 January to 9 April 2020

More cases were males (216, 56.7%) than females (165, 43.3%). Ages ranged between 1 year and 94 years with a mean age of 47.7 years (standard deviation, SD: 17.4; Fig. 3; Table 1). When stratified by infection source, a higher proportion of imported infections were in males but no difference by sex was seen for secondary and sporadic cases (Table 1). Only a small number of cases were in children regardless of infection source. A smaller proportion of patients were older than 70 years among the imported cases. Country of birth was available for 260 patients (68.2%), of whom the majority (191; 73.5%) were born in the United Kingdom. The ethnicity of the cases, available for 240 patients (63.0%), was comparable to the general population of England and Wales (Table 2).^18^

COVID-19: coronavirus disease 2019.

Note: Data on date of birth and sex were available for 381 cases of confirmed COVID-19.

**Table 1:** Age and sex distribution of COVID-19 cases in the First Few X study by case type in the United Kingdom of Great Britain and Northern Ireland, 31 January to 9 April 2020

Variable	No. (%) of COVID-19 cases		
	Total	Imported	Secondary or sporadic
**Sex**			
Male	216 (56.7)	123 (62.8)	93 (50.3)
Female	165 (43.3)	73 (37.2)	92 (49.7)
**Age, years**			
0–4	2 (0.5)	1 (0.5)	1 (0.5)
5–9	2 (0.5)	1 (0.5)	1 (0.5)
10–19	9 (2.4)	5 (2.6)	4 (2.2)
20–29	46 (12.1)	28 (14.3)	18 (9.7)
30–39	69 (18.1)	15 (7.7)	54 (29.2)
40–49	74 (19.4)	48 (24.5)	26 (14.1)
50–59	95 (24.9)	67 (34.2)	28 (15.1)
60–69	41 (10.8)	23 (11.7)	18 (9.7)
≥ 70	43 (11.3)	8 (4.1)	35 (18.9)
**All cases**	**381 (100.0)**	**196 (100.0)**	**185 (100.0) **

COVID-19: coronavirus disease 2019.

**Table 2:** Ethnicity of COVID-19 cases in the First Few X study compared with the general population of England and Wales, 31 January to 9 April 2020

Ethnic group^a^	No. (%) of people	
	COVID-19 cases	Population of England and Wales^b^
White	204 (85.0)	48 209 395 (86.0)
Asian, Asian British	15 (6.3)	4 213 531 (7.5)
Black, African, Caribbean, Black British	9 (3.8)	1 864 890 (3.3)
Other ethnic group	8 (3.3)	563 696 (1.0)
Mixed ethnicity	4 (1.7)	1 224 400 (2.2)
**All cases**	**240 (100.0)**	**56 075 912 (100.0)**

COVID-19: coronavirus disease 2019; United Kingdom: United Kingdom of Great Britain and Northern Ireland.

^a^ According to the Office for National Statistics of the United Kingdom.

^b^ From the 2011 Office for National Statistics census.^18^

Note: Data on ethnicity were available for 240 of the 381 confirmed cases of COVID-19. We were unable to ascertain the ethnicity of the cases in Scotland.

### Clinical features of cases

**Fig. 4:**
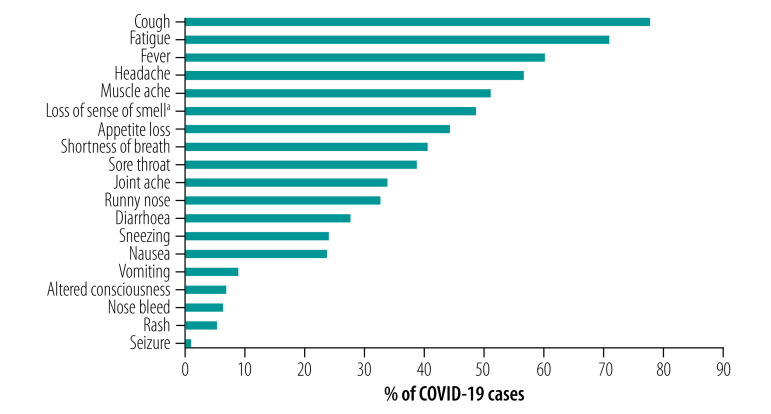
Symptoms reported over the course of illness among COVID-19 cases in the First Few X study in the United Kingdom of Great Britain and Northern Ireland, 31 January to 9 April 2020

The most frequent symptoms during illness were cough (296 cases; 77.7%), fatigue (270; 70.9%), fever (229; 60.1%), headache (216; 56.7%) and muscle ache (194; 50.9%). Of the 228 patients who reported whether their cough was dry or productive, the majority reported a dry cough (178; 78.1%). Anosmia was added to the follow-up questionnaire part way through the First Few X study. Nearly half of the 229 patients who were asked this question reported loss of sense of smell during their illness (111; 48.5%). One patient reported anosmia as their only symptom (Fig. 4).

COVID-19: coronavirus disease 2019.

^a^ Percentage of the 229 people with COVID-19 who were asked this question.

Notes: Data on symptoms were available for 381 cases of confirmed COVID-19. One case was asymptomatic.

Cough was the most common presenting symptom for all age groups. A lower proportion of patients in the ≥ 70 year old age group reported headache, sore throat, runny nose and sneezing compared with other age groups (data repository).^19^ Symptoms were relatively consistent comparing the sexes, although a higher proportion of the females than the males reported headache (103 patients; 62.4% versus 113 patients; 52.3%), sore throat (73 patients; 44.2% versus 74 patients; 34.3%), joint ache (62 patients; 37.6% versus 67 patients; 31.0%), diarrhoea (57 patients; 34.5% versus 48 patients; 22.2%) and nausea (52 patients; 31.5% versus 38 patients; 17.6%). Further data on presenting symptoms, including common groups (pairs and trios) of presenting symptoms, are available in the data repository.^19^

### Association of symptoms with COVID-19

Data on fever, cough, shortness of breath and sore throat were available both for 380 symptomatic COVID-19 cases using the First Few X protocol (one secondary case was asymptomatic, hence excluded) and for 752 contemporaneous confirmed non-COVID-19 respiratory infections. 

**Fig. 5:**
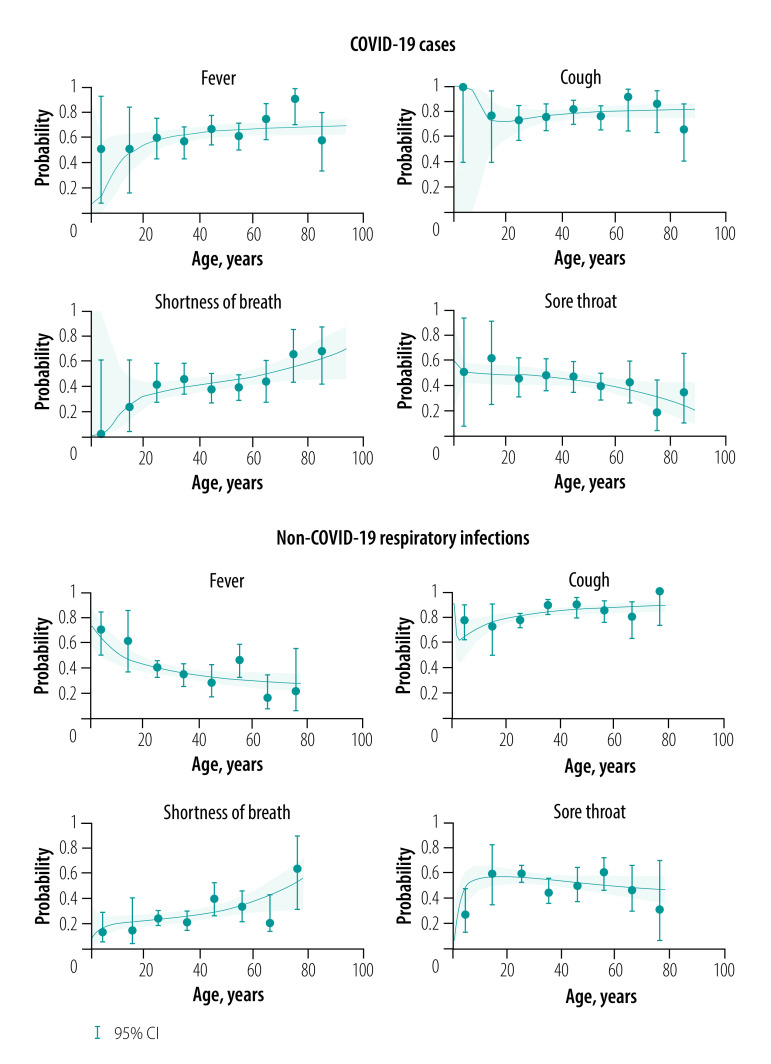
Relationship between symptoms and age in early COVID-19 positive and negative cases in the United Kingdom of Great Britain and Northern Ireland, 31 January to 9 April 2020

CI: confidence interval; COVID-19: coronavirus disease 2019.

Notes: We collected data on 380 people with symptomatic confirmed COVID-19 in the First Few X study and 752 people with confirmed non-COVID-19 respiratory infections in the minimum data set study at the start of the epidemic. The probability values are the proportion of cases in the respective age groups with that symptom.

The relationship between the presence of a symptom and age for people with COVID-19 and those with non-COVID-19 respiratory illness is nonlinear. For COVID-19 cases, there was an increasing occurrence of fever with increasing age, while for those with other respiratory infections the occurrence decreased with increasing age. For cough, a similar relationship was observed for COVID-19 and other respiratory infections. The occurrence of shortness of breath increased with increasing age for both groups, although in those with COVID-19 there was a higher proportion of elderly people with this symptom compared with those with other respiratory infections. The age relationship for sore throat was similar for both COVID-19 and other respiratory infections (Fig. 5).

Estimates of sensitivity and specificity for presence of symptoms in COVID-19 cases are presented in Table 3. We used broad age categories (< 30, 30–59 and ≥ 60 years) to provide estimates within these age strata. Fever had both good sensitivity and specificity (64.0% and 63.9%, respectively), cough had high sensitivity but poor specificity (79.6% and 15.5%, respectively), shortness of breath was the most specific symptom (75.5%) but had low sensitivity (42.1%), while sore throat had relatively low sensitivity and specificity (42.4% and 46.2%, respectively).

**Table 3:** Sensitivity and specificity of symptoms of COVID-19 in the First Few X study in the United Kingdom of Great Britain and Northern Ireland, 31 January to 9 April 2020

Symptom, by category	No. of COVID-19 cases with symptom/total no. of COVID-19 cases	No. of non-COVID-19 cases without symptom/total no. of non-COVID-19 cases	Sensitivity, % (95% CI)	Specificity, % (95% CI)
**Fever**				
All cases	226/353	418/654	64.0 (58.8–69.0)	63.9 (60.1–67.6)
Imported cases	108/177	418/654	61.0 (53.4–68.2)	NA
Sporadic cases	72/86	418/654	83.7 (74.2–90.8)	NA
Secondary cases	46/90	418/654	51.1 (40.3–61.8)	NA
Age < 30 years	30/52	166/279	57.7 (43.2–71.3)	59.5 (53.5–65.3)
Age 30–59 years	135/220	155/227	61.4 (54.6–67.8)	68.3 (61.8–74.3)
Age ≥ 60 years	61/81	35/41	75.3 (64.5–84.2)	85.4 (70.8–94.4)
**Cough**				
All cases	296/372	114/735	79.6 (75.1–83.6)	15.5 (13.0–18.3)
Imported cases	149/193	114/735	77.2 (70.6–82.9)	NA
Sporadic cases	75/86	114/735	87.2 (78.3–93.4)	NA
Secondary cases	72/93	114/735	77.4 (67.6–85.4)	NA
Age < 30 years	44/58	62/303	75.9 (62.8–86.1)	20.5 (16.1–25.4)
Age 30–59 years	183/233	27/264	78.5 (72.7–83.6)	10.2 (6.9–14.5)
Age ≥ 60 years	69/81	6/47	85.2 (75.6–92.1)	12.8 (4.8–25.7)
**Shortness of breath**				
All cases	154/366	500/662	42.1 (37.0–47.3)	75.5 (72.1–78.8)
Imported cases	54/191	500/662	28.3 (22.0–35.2)	NA
Sporadic cases	59/87	500/662	67.8 (56.9–77.4)	NA
Secondary cases	41/88	500/662	46.6 (35.9–57.5)	NA
Age < 30 years	20/57	224/277	35.1 (22.9–48.9)	80.9 (75.7–85.3)
Age 30–59 years	91/230	176/235	39.6 (33.2–46.2)	74.9 (68.8–80.3)
Age ≥ 60 years	43/79	32/45	54.4 (42.8–65.7)	71.1 (55.7–83.6)
**Sore throat**				
All cases	147/347	297/643	42.4 (37.1–47.8)	46.2 (42.3–50.1)
Imported cases	78/186	297/643	41.9 (34.8–49.4)	NA
Sporadic cases	28/74	297/643	37.8 (26.8–49.9)	NA
Secondary cases	41/87	297/643	47.1 (36.3–58.1)	NA
Age < 30 years	27/56	117/269	48.2 (34.7–62.0)	43.5 (37.5–49.6)
Age 30–59 years	97/224	112/231	43.3 (36.7–50.1)	48.5 (41.9–55.1)
Age ≥ 60 years	23/67	23/42	34.3 (23.2–46.9)	54.8 (38.7–70.2)
**Fever and/or cough**				
All cases	339/373	61/739	90.9 (87.5–93.6)	8.3 (6.4–10.5)
Imported cases	175/193	61/739	90.7 (85.7–94.4)	NA
Sporadic cases	82/88	61/739	93.2 (85.7–97.5)	NA
Secondary cases	82/92	61/739	89.1 (80.9–94.7)	NA
Age < 30 years	54/57	31/306	94.7 (85.4–98.9)	10.1 (7.0–14.1)
Age 30–59 years	211/234	15/266	90.2 (85.6–93.7)	5.6 (3.2–9.1)
Age ≥ 60 years	74/82	6/47	90.2 (81.7–95.7)	12.8 (4.8–25.7)
**Fever and cough**				
All cases	183/380	573/752	48.2 (43.0–53.3)	76.2 (73.0–79.2)
Imported cases	82/195	573/752	42.1 (35.0–49.3)	NA
Sporadic cases	65/91	573/752	71.4 (61.0–80.4)	NA
Secondary cases	36/94	573/752	38.3 (28.5–48.9)	NA
Age < 30 years	20/59	231/310	33.9 (22.1–47.4)	74.5 (69.3–79.3)
Age 30–59 years	107/237	210/268	45.1 (38.7–51.7)	78.4 (72.9–83.1)
Age ≥ 60 years	56/84	41/47	66.7 (55.5–76.6)	87.2 (74.3–95.2)

CI: confidence interval; COVID-19: coronavirus disease 2019; NA: not applicable.

^a^ We collected data on 380 symptomatic COVID-19 cases using the First Few X protocol and 752 contemporaneous confirmed non-COVID-19 respiratory infections. Data are missing in some categories.

Estimates of the positive predictive value and negative predictive value for the observed proportions of COVID-19 cases are presented in Table 4. The positive predictive value and negative predictive value were similar for fever and shortness of breath: 48.9% and 76.7%, respectively, for fever and 48.7% and 70.2%, respectively, for shortness of breath. The estimated positive predictive value increased with increasing age groups, with the negative predictive value decreasing.

**Table 4:** Predictive values of presenting symptoms of COVID-19 in the First Few X study in the United Kingdom of Great Britain and Northern Ireland, 31 January to 9 April 2020

Symptom, by category	No. of COVID-19 cases with symptom/ total with symptom	No. of non-COVID-19 cases without symptom/ total without symptom	Positive predictive value, % (95% CI)	Negative predictive value, % (95% CI)
**Fever**				
All cases	226/462	418/545	48.9 (44.3–53.6)	76.7 (72.9–80.2)
Imported cases	108/344	418/487	31.4 (26.5–36.6)	85.8 (82.4–88.8)
Sporadic cases	72/308	418/432	23.4 (18.8–28.5)	96.8 (94.6–98.2)
Secondary cases	46/282	418/462	16.3 (12.2–21.2)	90.5 (87.4–93.0)
Age < 30 years	30/143	166/188	21.0 (14.6–28.6)	88.3 (82.8–92.5)
Age 30–59 years	135/207	155/240	65.2 (58.3–71.7)	64.6 (58.2–70.6)
Age ≥ 60 years	61/67	35/55	91.0 (81.5–96.6)	63.6 (49.6–76.2)
**Cough**				
All cases	296/917	114/190	32.3 (29.3–35.4)	60.0 (52.7–67.0)
Imported cases	149/770	114/158	19.4 (16.6–22.3)	72.2 (64.5–79.0)
Sporadic cases	75/696	114/125	10.8 (8.6–13.3)	91.2 (84.8–95.5)
Secondary cases	72/693	114/135	10.4 (8.2–12.9)	84.4 (77.2–90.1)
Age < 30 years	44/285	62/76	15.4 (11.4–20.2)	81.6 (71.0–89.5)
Age 30–59 years	183/420	27/77	43.6 (38.8–48.5)	35.1 (24.5–46.8)
Age ≥ 60 years	69/110	6/18	62.7 (53.0–71.8)	33.3 (13.3–59.0)
**Shortness of breath**				
All cases	154/316	500/712	48.7 (43.1–54.4)	70.2 (66.7–73.6)
Imported cases	54/216	500/637	25.0 (19.4–31.3)	78.5 (75.1–81.6)
Sporadic cases	59/221	500/528	26.7 (21.0–33.0)	94.7 (92.4–96.4)
Secondary cases	41/203	500/547	20.2 (14.9–26.4)	91.4 (88.7–93.6)
Age < 30 years	20/73	224/261	27.4 (17.6–39.1)	85.8 (81.0–89.8)
Age 30–59 years	91/150	176/315	60.7 (52.4–68.5)	55.9 (50.2–61.4)
Age ≥ 60 years	43/56	32/68	76.8 (63.6–87.0)	47.1 (34.8–59.6)
**Sore throat**				
All cases	147/493	297/497	29.8 (25.8–34.1)	59.8 (55.3–64.1)
Imported cases	78/424	297/405	18.4 (14.8–22.4)	73.3 (68.7–77.6)
Sporadic cases	28/374	297/343	7.49 (5.0–10.6)	86.6 (82.5–90.0)
Secondary cases	41/387	297/343	10.6 (7.7–14.1)	86.6 (82.5–90.0)
Age < 30 years	27/179	117/146	15.1 (10.2–21.2)	80.1 (72.2–86.3)
Age 30–59 years	97/216	112/239	44.9 (38.2–51.8)	46.9 (40.4–53.4)
Age ≥ 60 years	23/42	23/67	54.8 (38.7–70.2)	34.3 (23.2–46.9)
**Fever and/or cough**				
All cases	339/1017	61/95	33.3 (30.4–36.6)	64.2 (53.7–73.8)
Imported cases	175/853	61/79	20.5 (17.9–23.4)	77.2 (66.4–85.9)
Sporadic cases	82/760	61/67	10.8 (8.7–13.2)	91.0 (81.5–96.6)
Secondary cases	82/760	61/71	10.8 (8.7–13.2)	85.9 (75.6–93.0)
Age < 30 years	54/329	31/34	16.4 (12.6–20.9)	91.2 (76.3–98.1)
Age 30–59 years	211/462	15/38	45.7 (41.1–50.3)	39.5 (24.0–56.6)
Age ≥ 60 years	74/115	6/14	64.3 (54.9–73.1)	42.9 (17.7–71.1)
**Fever and cough**				
All cases	183/362	573/770	50.6 (45.3–55.8)	74.4 (71.2–77.5)
Imported cases	82/261	573/686	31.4 (25.8–37.4)	83.5 (80.5–86.2)
Sporadic cases	65/244	573/599	26.6 (21.2–32.7)	95.7 (93.7–97.1)
Secondary cases	36/215	573/631	16.7 (12.0–22.4)	90.8 (88.3–92.9)
Age < 30 years	20/99	231/270	20.2 (12.8–29.5)	85.6 (80.0–89.5)
Age 30–59 years	107/165	210/340	64.8 (57.0–72.1)	61.8 (56.4–67.0)
Age ≥ 60 years	56/62	41/69	90.3 (80.1–96.4)	59.4 (46.9–71.1)

CI: confidence interval; COVID-19: coronavirus disease 2019; NA: not applicable; United Kingdom: United Kingdom of Great Britain and Northern Ireland.

^a^ We collected data on 380 symptomatic COVID-19 cases using the First Few X protocol and 752 contemporaneous confirmed non-COVID-19 respiratory infections. Data are missing in some categories.

After adjusting for the other symptoms, age and sex, two symptoms were significantly associated with a diagnosis of COVID-19: fever (adjusted odds ratio, OR: 4.15; 95% confidence interval, CI: 2.95–5.82) and shortness of breath (adjusted OR: 2.27; 95% CI: 1.56–3.29). Cough and sore throat did not have a significant association with COVID-19 (adjusted OR: 0.73; 95% CI: 0.48–1.09 and adjusted OR: 0.78; 95% CI: 0.56–1.10, respectively; Table 5).

**Table 5:** Association between COVID-19 diagnosis and symptoms, age and sex in the First Few X study in the United Kingdom of Great Britain and Northern Ireland, 31 January to 9 April 2020

Variable	No. of people positive/negative for COVID-19^a^	Single variable analysis, OR (95% CI)	Multivariable analysis, adjusted OR (95% CI)^b^
Fever	226/236	3.15 (2.41–4.13)	4.15 (2.95–5.82)
Cough	296/621	0.71 (0.52–0.99)	0.73 (0.48–1.09)
Shortness of breath	154/162	2.24 (1.71–2.95)	2.27 (1.56–3.29)
Sore throat	147/346	0.63 (0.48–0.82)	0.78 (0.56–1.10)
Age (10 years)	NA	1.65 (1.52– 1.79)	1.63 (1.47–1.81)
Male	216/349	1.45 (1.13–1.85)	1.26 (0.90–1.76)

CI: confidence interval; COVID-19: coronavirus disease 2019; OR: odds ratio; NA: not applicable.

^a^ We collected data on 380 symptomatic COVID-19 cases using the First Few X protocol and 752 contemporaneous confirmed non-COVID-19 respiratory infections.

^b^ Adjusted for other symptoms, age and sex.

As the occurrence of symptoms changed with age, we explored interaction terms between a symptom and broader age groups. This analysis provided strong evidence that the association with fever differed in these age groups (interaction term: *P* < 0.001). The adjusted OR was 2.67 (95% CI: 1.41–5.07) in the under 30-year-olds, 4.08 (95% CI: 2.60–6.41) in the 30–59-year-olds and 17.15 (95% CI: 5.60–52.55) in those ≥ 60 years of age. No other symptom exhibited any strong evidence of an interaction with age. We found similar results when applying a multinomial regression model (data repository).^19^

### Health-care interactions and clinical course

We obtained overall follow-up information on 338 of the 381 of the included cases (88.7%). Among the 154 patients with sufficient recorded information, the duration of illness ranged from 2 to 36 days (median: 11 days, interquartile range: 7–15 days; mean: 12.1 days). 

The most common health-care interaction among the 359 cases in England was use of the government’s telephone and online service (called NHS 111), with just over three quarters of patients accessing the service at least once (Box 2). Smaller proportions of patients visited their general practitioner (47; 13.1%) or accident and emergency department (103; 28.7%). A total of 154 patients (42.9%) were hospitalized, of whom 35 were admitted to intensive care units (22.7% of those hospitalized) and 25 required mechanical ventilation (16.2% of those hospitalized).

Box 2 **Health-care interactions and clinical course of COVID-19 cases in the First Few X study, England, 31 January to 9 April 2020**

Used telephone or online helpline^a^: 277 cases (77.2%).

Visited general practitioner: 47 cases (13.1%).

Visited accident and emergency department: 103 cases (28.7%).

Hospitalized: 154 cases (42.9%).

Admitted to intensive care unit: 35 cases (9.7%).

Received mechanical ventilation: 25 cases (7.0%).

Diagnosed with acute respiratory distress syndrome: 12 cases (3.3%).

Having chest X-ray evidence of pneumonia: 57 cases (15.9%).

Received extracorporeal membrane oxygenation: 1 case (0.3%).

COVID-19: coronavirus disease 2019. ^a^ The helpline is called NHS 111.Note: Data on health-care interactions and clinical course were obtained for 359 of 381 confirmed cases of COVID-19.

We ascertained clinical outcomes for 302 of these patients. At the time of follow-up 220 (72.8%) of these patients had recovered from their COVID-19 illness, 57 (18.9%) were still ill (self-reported illness) and 25 (8.3%) had died.

## Discussion

This study presents an early assessment of the epidemiological and clinical characteristics of COVID-19 patients in the United Kingdom. Just over half of the cases included in our study were imported, the majority from Italy, highlighting the importance of the Italian outbreak in facilitating spread to other European countries.^20^ Sporadic cases were detected within a month of the first confirmed case in the United Kingdom. The age and sex distribution of First Few X cases were similar to those described elsewhere.^4^^–^^7^^,^^10^^,^^12^ Only a small proportion of patients were children; an age distribution that was described in early cases series,^4^^–^^7^ and continues to be seen in the United Kingdom up to November 2020.^21^ The ethnic distribution of non-white ethnic groups being disproportionately affected by COVID-19 that has become evident in the United Kingdom population^21^^,^^22^ was not apparent among the first cases. The clinical presentation in COVID-19-infected patients was dominated by cough, fatigue and fever, consistent with other studies.^4^^–^^7^^,^^9^^,^^10^^,^^12^ Almost half of patients reported anosmia during their illness, a symptom that was later added to the United Kingdom’s COVID-19 symptom list.^23^

We noted differences in symptom presentation by age, with a lower proportion of patients in the youngest (< 20 years) and oldest age groups (≥ 70 years) reporting symptoms when compared with the other age groups. In particular, a nonlinear relationship with age was observed for fever which increased with age for the COVID-19 cases. Although this study included only a small number of children, our findings are congruent with other studies suggesting that children may experience milder illness with different symptoms.^24^^–^^26^ The different sensitivity and specificity of symptoms by age highlights the need to consider age when setting up case definitions to support public health risk assessment, clinical triage and diagnostic algorithms.

Fever was clearly an important symptom, exhibiting good sensitivity and specificity. This symptom was also significantly associated with a diagnosis of COVID-19, as was shortness of breath, and there was evidence of an interaction between fever and age, including for the different case types. Notably, although cough was a common symptom among COVID-19 patients, it had lower specificity, also being common among people testing negative for COVID-19, and was not significantly associated with a confirmed COVID-19 diagnosis.

The non-urgent medical telephone and online service was the most common health-care interaction of the study patients, in keeping with key government messaging during the study period which emphasized that those experiencing COVID-19 symptoms should stay home and contact the service. This finding highlights the importance of public messaging about using online and telephone services to avoid propagating transmission of COVID-19 in health-care settings. Forty-three per cent of patients included in our study were hospitalized. However, this is certainly an overestimate of the case hospitalization rate since at the beginning of the country’s COVID-19 incident response all confirmed cases were hospitalized for isolation rather than clinical management purposes, and from 13 March 2020, testing was restricted to hospitalized cases only. The finding that only a small proportion of those hospitalized patients required mechanical ventilation in comparison with other studies supports this.^4^^,^^6^

Using the WHO First Few X Unity protocol and by adapting pandemic influenza protocols and systems, we were able to systematically collect detailed epidemiological and clinical data. Initially local health protection teams undertook the data collection, achieving high completion rates for the initial case forms, however due to the rapid increase in case numbers and challenges around public health management of patients, there were capacity constraints in following up cases and contacts. Ultimately, Public Health England established a large dedicated team to undertake case and contact follow-up and the study achieved high rates of follow-up on the First Few X cases. These actions highlight the challenges for countries attempting to implement First Few X studies in the context of a large pandemic and we would recommend that this type of study is conducted by a dedicated team, separate from those responsible for the public health management of cases. Despite having a high follow-up rate, clinical outcome was not available for some patients due to sensitivities and difficulties around ascertaining the required information, particularly for the most severely ill people.

A further limitation of the study is that the included cases are likely to be biased towards more severely ill people who presented to health care, and they will therefore under-represent those with mild illness in the population. The clinical presentation of First Few X cases may also differ from that of all United Kingdom cases since the imported cases (accounting for more than half of all cases) were more likely to have been of working age and may have been healthier than the general population. The severity estimates are also likely to be overestimates due to policy changes over the course of the study period, with hospitalization of patients for isolation purposes initially and latterly restricting testing to hospitalized patients only. The analyses using data from those testing negative for COVID-19 were likely to be influenced by the testing criteria at the time of the study, and the positive predictive value dependent on the infection prevalence which would have been low at the time of the study but rapidly increasing in some parts of the country. These analyses were also limited by only having four symptoms collected on the possible cases via the minimum data set forms. Furthermore, recent studies that include serology and polymerase chain reaction tests indicate that a large proportion of COVID-19 cases are missed by this test, especially if the test is done more than 9 days after symptom onset. We may therefore have underestimated the number of cases.^27^

Future pandemic planning should note the importance of maintaining First Few X studies into the community transmission phase, as we have shown differences between imported and United Kingdom-acquired cases of COVID-19. Furthermore, achieving high quality and complete data capture and follow-up of cases depends on the ability to rapidly mobilize a cadre of trained public health professionals with sufficient resources to interview cases, clinicians and contacts. This mobilization can pose a challenge when capacity is already overwhelmed by the incident response. Consideration should also be given to case ascertainment through First Few X investigations and how this may differ as a pandemic progresses due to changing contact tracing and testing policies over time.
